# Baseline Fasting Glucose Level, Age, Sex, and Body Mass Index and the Development of Diabetes in US Adults

**DOI:** 10.1001/jamanetworkopen.2024.56067

**Published:** 2025-01-23

**Authors:** Aoife M. Egan, Christina M. Wood-Wentz, Sneha Mohan, Kent R. Bailey, Adrian Vella

**Affiliations:** 1 Division of Endocrinology, Diabetes and Metabolism, Mayo Clinic, Rochester, Minnesota; 2 Division of Clinical Trials and Biostatistics, Mayo Clinic, Rochester, Minnesota

## Abstract

**Importance:**

Understanding the interplay between diabetes risk factors and diabetes development is important to develop individual, practice, and population-level prevention strategies.

**Objective:**

To evaluate the progression from normal and impaired fasting glucose levels to diabetes among adults.

**Design, Setting, and Participants:**

This retrospective community-based cohort study used data from the Rochester Epidemiology Project, in Olmsted County, Minnesota, on 44 992 individuals with at least 2 fasting plasma glucose (FPG) measurements from January 1, 2005, to December 31, 2017. People who met criteria for diabetes on or before their first FPG measurement were excluded. Data were electronically retrieved in December 2019 with analyses finalized in November 2024.

**Exposures:**

The exposure was baseline FPG level, with covariates including the following measures that are consistently recorded in the electronic health record: body mass index (BMI), age, and sex.

**Main Outcomes and Measures:**

The cumulative probability of freedom from diabetes was estimated and presented graphically using a Kaplan-Meier curve. Multivariable Cox proportional hazards regression modeling was used to estimate the partial hazard ratios (HRs) for variables of interest. Diabetes was defined as an FPG level greater than 125 mg/dL.

**Results:**

A total of 44 992 individuals (mean [SD] age at baseline, 43.7 [11.8] years; 26 025 women [57.8%]) were included. The baseline mean (SD) BMI was 28.9 (6.6). Over a median follow-up of 6.8 years (IQR, 3.6-9.7 years), 3879 individuals (8.6%) developed diabetes. The Kaplan-Meier 10-year cumulative risk of incident diabetes was 12.8% (95% CI, 12.4%-13.2%). All initial FPG levels outside a range of 80 to 94 mg/dL were associated with increased risk for diabetes (ie, FPG <70 mg/dL: HR, 3.49 [95% CI, 2.19-5.57]; FPG 120-125 mg/dL: HR, 12.47 [10.84-14.34]). Other independent risk factors were male sex (HR, 1.31 [95% CI, 1.22-1.40]), older age (≥60 years: HR, 1.97 [95% CI, 1.71-2.28]), and any abnormal category of BMI, including underweight (BMI <18.5: HR, 2.42 [95% CI, 1.77-3.29]; BMI ≥40: HR, 4.03 [95% CI, 3.56-4.56]). There was a significant additive association of variables, particularly FPG level and BMI. For instance, a woman aged 55 to 59 years with a BMI of 18.5 to 24.9 and an FPG level of 95 to 99 mg/dL had an estimated 10-year diabetes risk of 7.0%. However, an almost doubling of risk to 13.0% was observed if the BMI was 30.0 to 34.9, and risk more than doubled again to 28.0% if FPG level also increased to 105 to 109 mg/dL. A nomogram was generated to facilitate individual classification into one of four 10-year risk categories.

**Conclusions and Relevance:**

This retrospective cohort study of 44 992 individuals suggests that FPG level, age, BMI, and male sex were all associated with development of diabetes, with significant interaction between these variables. These data contribute to understanding the clinical course of diabetes and highlight the substantial individual variation in diabetes risk according to commonly measured clinical variables. The findings facilitate lifestyle and pharmacologic interventions to treat those at highest risk of diabetes to reduce future morbidity and mortality. Further work is needed to validate this risk categorization tool for different populations.

## Key Points

**Question:**

Is fasting plasma glucose (FPG) level in combination with anthropometric variables associated with a 10-year risk of diabetes?

**Findings:**

In this cohort study of 44 992 individuals, the 10-year cumulative risk of incident diabetes was 12.8%. Fasting plasma glucose level, body mass index, older age, and male sex were associated with diabetes development, with significant interaction among these variables.

**Meaning:**

These data facilitate identification and treatment of those who are at highest risk of diabetes development despite having a normal FPG level.

## Teaser

This cohort study evaluates the association of fasting plasma glucose levels in combination with anthropometric variables with 10-year risk of diabetes among adults.

## Introduction

The transition from normal fasting glucose level and normal glucose tolerance to type 2 diabetes is typically characterized by an intermediate state of impaired fasting glucose and/or impaired glucose tolerance, also termed *prediabetes*. Lifestyle and pharmacologic interventions can reduce the incidence of diabetes among individuals at high risk.^1,2^ The American Diabetes Association recommends conducting a clinical risk factor assessment to guide whether to perform a diagnostic test for prediabetes or diabetes.^3^ Diagnostic testing is based on plasma glucose criteria, either the fasting plasma glucose (FPG) value or the 2-hour plasma glucose value during a 75-g oral glucose tolerance test or the hemoglobin A_1c_ (HbA_1c_) level.^4,5^

Large cohorts with access to clinical data are critical to our understanding of the clinical course of diabetes and enable tailored prevention strategies to reduce long-term morbidity and mortality.^6^ The Rochester Epidemiology Project (REP) facilitates population-based research in Olmsted County, Minnesota, using a medical record linkage system. A prior study using REP data from 1983 to 1995 observed that 10.3% of residents aged 40 years or older developed diabetes after a median follow-up of 9 years (range, 0-13 years).^7^ They found that the FPG value was a major determinant of an individual’s subsequent risk of developing diabetes, even among individuals whose FPG level was within the normal range at baseline. However, the cumulative association of additional variables with diabetes risk was not assessed, and the study predated the obesity pandemic, which significantly increased the national prevalence of diabetes.^8^ In addition, in the group with the highest 10-year risk of diabetes development (FPG ≥110 mg/dL [to convert to millimoles per liter, multiply by 0.0555]), only 38.6% progressed to diabetes, suggesting that factors beyond FPG level contribute to the progression of prediabetes to diabetes.

We therefore designed a study to ascertain the 10-year cumulative risk of incident diabetes in a contemporary REP cohort containing individuals who had normal or impaired fasting glucose levels at baseline. We constructed models for this 10-year risk as a function of age, sex, body mass index (BMI; calculated as weight in kilograms divided by height in meters squared), and initial level of FPG, including interactions among these variables, which tend to be consistently recorded in outpatient encounters. Data were then used to construct a nomogram to provide a single numerical estimate of individual risk.

## Methods

### Study Design and Population

The REP links medical records across several medical organizations to identify unique persons (ie, a records-linkage system). From this system, the REP constructs personal residency timelines, allowing enumeration of virtually all persons who have resided in Olmsted County, Minnesota, from January 1, 1966, to the present.^9^ Electronic laboratory test data are available from 2005 onward. Details on overall population size, demographics, and socioeconomic status have been previously published.^10^ In this retrospective cohort study, the REP resource was used to identify a cohort of individuals aged 18 to 65 years living in Olmsted County with at least 2 FPG tests between January 1, 2005, and December 31, 2017 (eFigure 1 in Supplement 1). Included glucose samples were restricted to specific ordering codes to maximize the chances of including only fasting samples, an approach used in previous REP publications.^11^ Admission and discharge dates were used to exclude samples taken during an inpatient hospitalization. Individuals with a preexisting diagnostic code for diabetes, those prescribed a glucose-lowering medication, and those with an FPG level of 126 mg/dL or more on or before their first FPG measurement were excluded. Source data were manually examined in a subset of 620 individuals who had an FPG level less than 80 mg/dL, which confirmed accuracy of the electronically retrieved data in all cases. The project was approved by the institutional review boards of Mayo Clinic, Rochester, Minnesota, and the Olmsted Medical Center. As required by Minnesota law, individuals included in the REP do not need to provide written informed consent for a specific study, but they must authorize the use of their data for research before researchers can review their health record. According to previous studies, 98.0% of individuals gave authorization to use their data for research.^12^ We followed the Reporting of Studies Conducted Using Observational Routinely-Collected Health Data (RECORD) statement on the reporting of observational studies that use routinely collected health data.^13^

### Diagnosis of Diabetes

Diabetes was diagnosed in those with an FPG level of 126 mg/dL or more. Those who developed diabetes were assigned an event date as the date of their first diagnostic value. Individuals who did not meet the criterion for diabetes were censored on the date of their last FPG value on or before the earlier of December 31, 2017, or death.

### Statistical Analysis

Data were electronically retrieved in December 2019 with analyses finalized in November 2024. Descriptive statistics for the entire cohort of 44 992 individuals, and subdivided by incident diabetes, included mean (SD) values for continuous variables and counts and percentages for discrete variables. The cumulative probability of freedom from diabetes (conditional on survival) was estimated and presented graphically using the Kaplan-Meier method. The variables of interest were age, sex, BMI, and initial level of FPG. Diabetes risk was analyzed according to 6 categories of baseline BMI (underweight, <18.5; normal weight, 18.5-24.9; overweight, 25.0-29.9; class I obesity, 30.0-34.9; class II obesity, 35.0-39.9; and class III obesity, ≥40.0); 9 categories of initial FPG level (<70, 70-79, 80-94, 95-99, 100-104, 105-109, 110-114, 115-119, and 120-125 mg/dL); 4 categories of baseline age (18-29.9, 30-54.9, 55-59.9, and 60-65 years); and sex (male or female). The categories for BMI were based on Centers for Disease Control and Prevention definitions,^14^ while the other categories were based on a selection process that started with grids of 5 years for age and 5 mg/dL for FPG level. The selection process required that each step for a given variable be associated with a significant improvement in fit, or we collapsed it with other levels. The resulting set of dichotomous variables was used in multivariable Cox proportional hazards regression modeling to estimate the partial hazard ratios (HRs) and 95% CIs for each category of baseline BMI, FPG level, age, and sex. Namely, we fit an additive (main associations) multiple variable model but also tested for 2-way interactions between pairs of dichotomous variables.

We used the no-interaction model to calculate the estimated risk of any individual relative to the risk of an individual having the lowest possible risk. The goodness of fit of the models was tested using the Hosmer-Lemeshow method, by defining deciles of risk as derived from each model and comparing the Kaplan-Meier estimates of 10-year risk with the model-based estimated mean risk within each bin. In addition, by examining the coefficients in the Cox proportional hazards multiple regression model, we developed a scoring system to reproduce this relative risk and used it to construct a nomogram to depict individual risk. Sensitivity analyses were conducted for the 4-category age model by investigating a model using age + age squared. The differences were found to be minor but in favor of the categorical age model (eAppendix in Supplement 1).

Statistical analyses were completed using SAS, version 9.4 (SAS Institute Inc). All *P* values were from 2-sided tests, and results were deemed statistically significant at *P* < .05.

## Results

**Figure 1.**
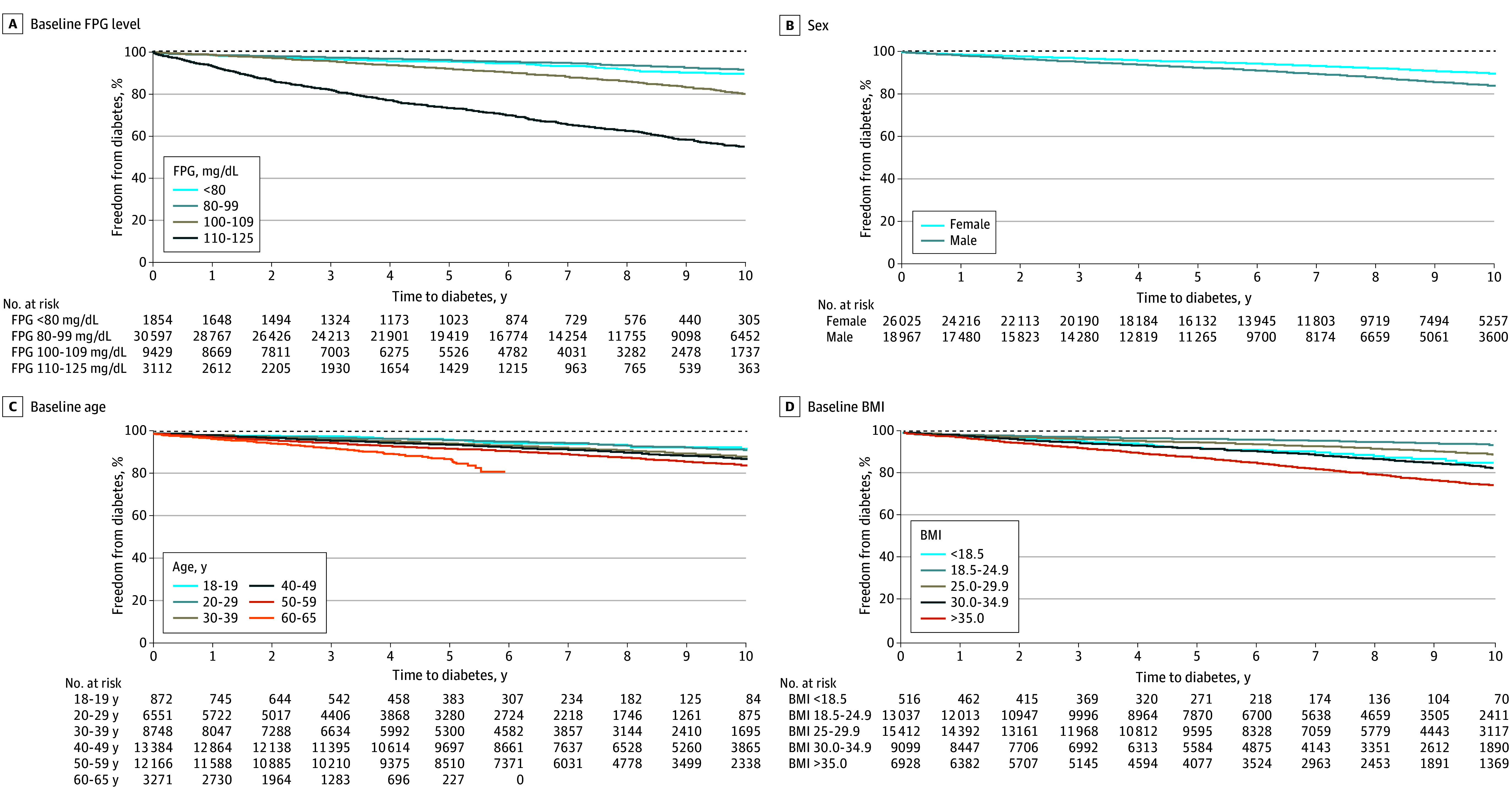
Ten-Year Cumulative Freedom From Incident Diabetes According to Baseline Category of Fasting Plasma Glucose (FPG) Level, Sex, Baseline Age, and Body Mass Index (BMI) Body mass index is calculated as weight in kilograms divided by height in meters squared. To convert FPG to millimoles per liter, multiply by 0.0555.

In total, 44 992 individuals (mean [SD] age at baseline, 43.7 [11.8] years; age range, 18-65 years; 26 025 women [57.8%] and 18 967 men [42.2%]; 1844 Asian participants [4.1%], 1934 Black participants [4.3%], 39 178 White participants [87.1%], and 2036 participants of other race or ethnicity [4.5%]; mean [SD] BMI, 28.9 [6.6]) were eligible for inclusion in the cohort. Table 1 summarizes the baseline characteristics of all individuals as a group and according to diabetes status at study completion. The proportional hazards assumption was met. Over a median follow-up of 6.8 years (IQR, 3.6-9.7 years), 3879 individuals (8.6%) developed diabetes: 7.1% of all women (1847 of 26 025) and 10.7% of all men (2032 of 18 967). The Kaplan-Meier 10-year cumulative risk of incident diabetes was 12.8% (95% CI, 12.4%-13.2%). The 10-year cumulative freedom from diabetes is presented according to baseline category of FPG, sex, age, and BMI in Figure 1.

**Table 1.** Baseline Characteristics of All Individuals Included in the Cohort and According to Diabetes Development

Characteristic	Individuals, No. (%)		
	Total (N = 44 992)	Did not develop diabetes (n = 41 113 [91.4%])	Developed diabetes (n = 3879 [8.6%])
Sex			
Female	26 025 (57.8)	24 178 (58.8)	1847 (47.6)
Male	18 967 (42.2)	16 935 (41.2)	2032 (52.4)
Race and ethnicity			
Asian	1844 (4.1)	1653 (4.0)	191 (4.9)
Black	1934 (4.3)	1715 (4.2)	219 (5.7)
White	39 178 (87.1)	35 934 (87.4)	3244 (83.6)
Other^a^	2036 (4.5)	1811 (4.4)	225 (5.8)
Age, mean (SD), y	43.7 (11.8)	42.9 (12.0)	45.6 (10.0)
Age category, y			
<30.0	7422 (16.5)	7096 (17.2)	326 (8.4)
30.0-54.9	29 115 (64.7)	26 312 (64.0)	2803 (72.3)
55.0-59.9	5184 (11.5)	4654 (11.3)	530 (13.7)
≥60.0	3271 (7.3)	3051 (7.4)	220 (5.7)
BMI, mean (SD)	28.9 (6.6)	28.5 (6.4)	32.4 (7.5)
BMI			
<18.5	516 (1.2)	472 (1.2)	44 (1.1)
18.5-24.9	13 037 (29.0)	12 540 (30.5)	497 (12.8)
25.0-29.9	15 412 (34.3)	14 358 (34.9)	1054 (27.1)
30.0-34.9	9099 (20.2)	8055 (19.6)	1044 (27.0)
35.0-39.9	4100 (9.1)	3418 (8.3)	682 (17.6)
≥40.0	2828 (6.3)	2270 (5.5)	558 (14.4)
FPG, mean (SD), mg/dL	94.7 (9.5)	94.0 (9.1)	101.2 (11.6)
FPG, mg/dL			
<80	1854 (4.1)	1742 (4.2)	112 (2.9)
80-99	30 597 (68.0)	28 976 (70.5)	1621 (41.8)
100-109	9429 (21.0)	8241 (20.0)	1188 (30.6)
110-125	3112 (6.9)	2154 (5.2)	958 (24.7)

Abbreviations: BMI, body mass index (calculated as weight in kilograms divided by height in meters squared); FPG, fasting plasma glucose.

SI conversion factor: To convert glucose to millimoles per liter, multiply by 0.0555.

^a^ Includes the following categories: American Indian, Hawaiian or Pacific Islander, other or mixed, refused to answer, and unknown.

Results from the Cox proportional hazards regression models identified significant risk associations with all examined variables (Table 2). Reduced or elevated initial FPG level was associated with increased risk compared with a reference FPG group of 80 to 94 mg/dL (ie, FPG <70 mg/dL: HR, 3.49 [95% CI, 2.19-5.57]; FPG 120-125 mg/dL: HR, 12.47 [10.84-14.34]). Male sex was associated with increased risk compared with female sex (HR, 1.31 [95% CI, 1.22-1.40]). Any abnormal BMI category was associated with increased risk compared with a reference BMI category of 18.5 to 24.9 (BMI <18.5: HR, 2.42 [95% CI, 1.77-3.29]; BMI ≥40: HR, 4.03 [95% CI, 3.56-4.56]). Finally, increasing age was also associated with increased risk of diabetes development (≥60 years: HR, 1.97 [95% CI, 1.71-2.28]).

**Table 2.** Univariate and Multivariable Cox Proportional Hazards Regression Analyses for Risk of Diabetes Development

Factor	HR (95% CI)	
	Univariate	Multivariable
Sex		
Female	1 [Reference]	1 [Reference]
Male	1.56 (1.47-1.66)	1.31 (1.22-1.40)
Age category, y		
<30.0	0.64 (0.57-0.72)	0.74 (0.66-0.83)
30.0-54.9	1 [Reference]	1 [Reference]
55.0-59.9	1.48 (1.35-1.63)	1.33 (1.21-1.47)
≥60.0	2.29 (1.99-2.64)	1.97 (1.71-2.28)
BMI		
<18.5	2.57 (1.89-3.50)	2.42 (1.77-3.29)
18.5-24.9	1 [Reference]	1 [Reference]
25.0-29.9	1.73 (1.55-1.92)	1.36 (1.22-1.52)
30.0-34.9	2.91 (2.61-3.24)	2.11 (1.89-2.35)
35.0-39.9	4.28 (3.81-4.81)	3.22 (2.86-3.62)
≥40.0	5.34 (4.74-6.03)	4.03 (3.56-4.56)
FPG, mg/dL		
<70	2.71 (1.70-4.32)	3.49 (2.19-5.57)
70-79	1.37 (1.11-1.69)	1.58 (1.28-1.95)
80-94	1 [Reference]	1 [Reference]
95-99	1.49 (1.35-1.65)	1.29 (1.16-1.42)
100-104	2.48 (2.25-2.74)	2.00 (1.81-2.21)
105-109	3.82 (3.43-4.24)	2.94 (2.64-3.28)
110-114	6.14 (5.46-6.91)	4.51 (4.00-5.09)
115-119	11.22 (9.89-12.72)	8.05 (7.08-9.16)
120-125	16.85 (14.68-19.34)	12.47 (10.84-14.34)

Abbreviations: BMI, body mass index (calculated as weight in kilograms divided by height in meters squared); FPG, fasting plasma glucose; HR, hazard ratio.

SI conversion factor: To convert glucose to millimoles per liter, multiply by 0.0555.

Interactions were assessed, and although a couple of them were nominally significant due to the power of this large study, they were deemed to be of minimal clinical importance. Therefore, attention was focused on the additive model and its interpretability through a nomogram for clinician utility in patient shared decision-making.

**Figure 2.**
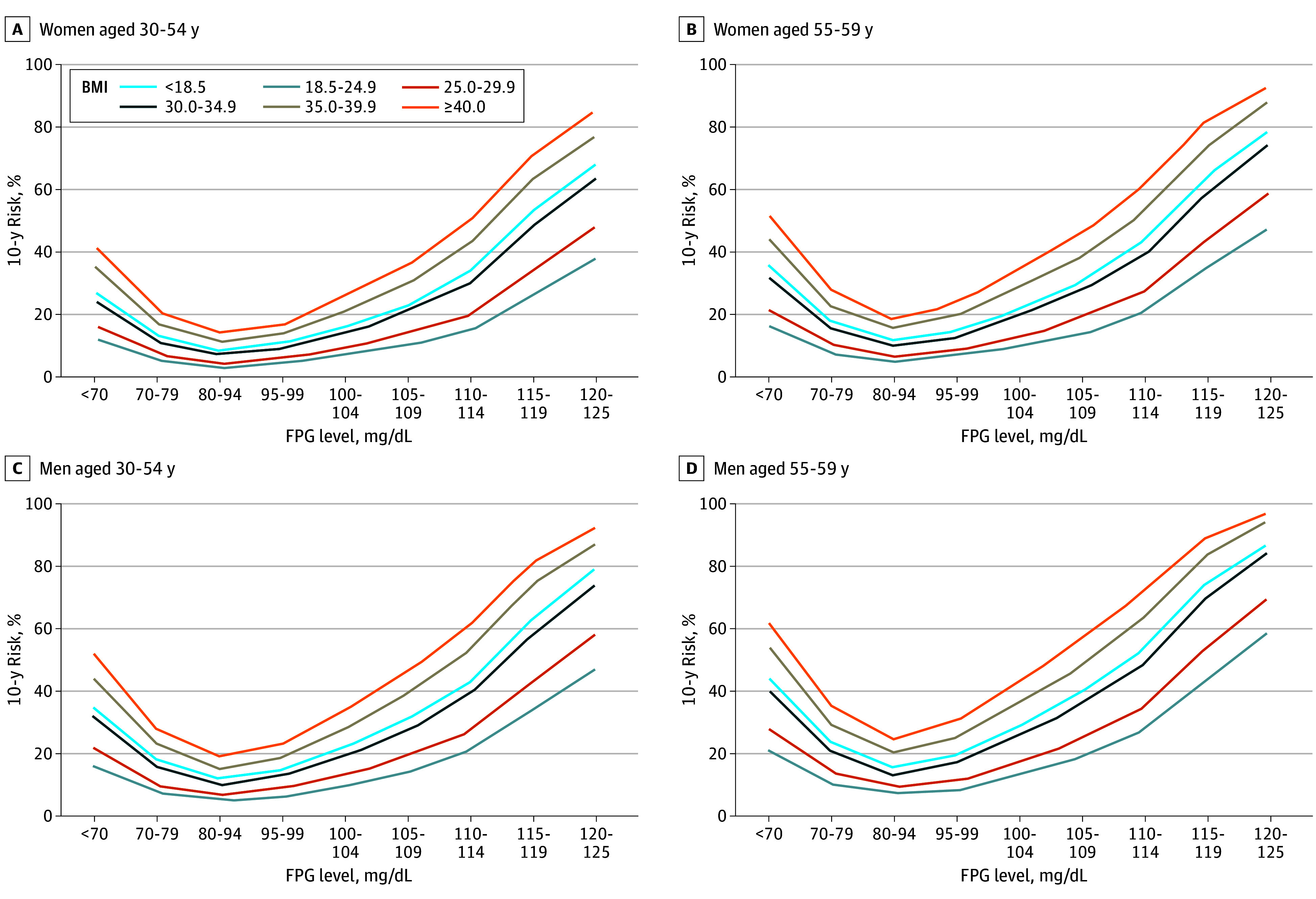
Ten-Year Risk Across Baseline Fasting Plasma Glucose (FPG) Concentrations, by Body Mass Index (BMI) Groups for Women and Men, According to Age Category Body mass index is calculated as weight in kilograms divided by height in meters squared. To convert FPG to millimoles per liter, multiply by 0.0555.

The 10-year risk tables for our additive model are in eTable 1 in Supplement 1. Figure 2 and eFigures 2 and 3 in Supplement 1 provide a graphical representation of these data. In Figure 2 and eFigure 2 in Supplement 1, the 10-year risk is presented across increasing categories of FPG level, according to BMI and age categories for women and men. eFigure 3 in Supplement 1 presents risk across BMI groups according to categories of age and FPG level for women and men. In this way, the additive association of these variables is evident. For example, a woman aged 55 to 59 years with a BMI of 18.5 to 24.9 and an FPG level of 95 to 99 mg/dL had an estimated 10-year diabetes risk of 7.0%. However, an almost doubling of risk to 13.0% was observed if the BMI was 30.0 to 34.9, and risk more than doubled again to 28.0% if the FPG level was also increased to 105 to 109 mg/dL. Across all age groups, both men and women with a BMI less than 18.5 or an FPG level below 80 to 94 mg/dL were at increased risk compared with their counterparts in the lowest risk category for the respective variable. Although increasing categories of FPG level above 80 to 94 mg/dL were associated with a steady increase in risk of diabetes, a sharp inflection is not noted at 100 mg/dL, the cutoff frequently used to define prediabetes.^5^ The results of the Hosmer-Lemeshow comparison of estimated 10-year risk with the risk obtained from the Kaplan-Meier method within deciles of risk are shown in eTable 2 and eFigure 4 in Supplement 1, which shows a plot of model-estimated risk vs empirical risk. Other than the very lowest risk decile, the points fall almost perfectly on the line of identity. In the lowest risk decile, the additive model slightly overestimates the empirical risk. Although this deviation is statistically significant, it is slight and in the direction of overstating, not understating, risk.

The nomogram (eFigure 5 in Supplement 1) uses the lowest risk category for each variable as a reference and assigns a specific number of points to each category of FPG level, age, sex, and BMI. The cumulative points score for all the variables can range from 0 to 17 and can then be categorized into one of four 10-year risk categories: minimal (0-3 points), low (4-6 points), moderate (7-9 points), and high (≥10 points). Each category is associated with an incremental increase in 10-year risk of diabetes: minimal, 5% (95% CI 5%-6%); low, 12% (12%-13%); moderate, 26% (24%-27%); and high, 56% (52%-59%). Corresponding Kaplan-Meier curves are provided in eFigure 6 in Supplement 1.

## Discussion

In this cohort study of individuals without diabetes at baseline, we found that over a median follow-up of 6.8 years, 8.6% developed diabetes, and the 10-year cumulative risk of incident diabetes was 12.8%. Risk increased significantly as baseline FPG level increased, even within the accepted normal range. This finding is in keeping with other studies assessing FPG level, several of which focused on participants with prediabetes at baseline.^15,16,17,18,19^ We also demonstrated the additive association of key additional variables, including male sex, increasing age, and BMI. The nomogram provides a single risk estimate for a given individual over the subsequent 10 years considering the relative contribution of each variable. This risk estimate allows for identification of individuals with the highest risk and can facilitate options for tailored intervention.

The higher risk for diabetes development among individuals across both sexes and all age groups with an underweight BMI or an FPG concentration less than 80 mg/dL at baseline is interesting but biologically plausible, as there is a known curvilinear association between many biological parameters (including glucose) and health outcomes.^20,21,22^ It is possible that underweight BMI and lower FPG level are markers of poor nutritional status and overall health. Skeletal muscle is essential for glucose clearance, and sarcopenia is associated with impaired insulin action and diabetes development.^23^ However, sarcopenia is not easily and reproducibly captured during routine clinical examination or in the medical record. We did not discriminate based on type of diabetes. However, given the relatively low annual incidence of type 1 diabetes of 9.2 per 100 000 people per year (all ages) in Olmsted County,^24^ and the fact that we excluded individuals younger than 18 years of age, we anticipate that most individuals in this study who progressed to diabetes had a diagnosis of type 2 diabetes.

With regard to choice of diagnostic test, measuring the FPG level has the advantage of being widely available and inexpensive, but the associated fasting requirement is burdensome, and the test is associated with significant biological variation.^25^ There is an increasing move toward the use of HbA_1c_ level, or a combination of FPG level and HbA_1c_ level for diagnosis of diabetes.^5,26^ Using the HbA_1c_ level overcomes many of the disadvantages of using the FPG level, but it is more expensive, not as widely available, and may be influenced by factors other than glucose, including race and ethnicity and change in erythrocyte life span.^25^ Discriminating based on FPG level vs HbA_1c_ level will detect different populations,^27^ and these tests individually and combined are less sensitive and specific than the criterion standard oral glucose tolerance test.^28^ In a study using data from 117 worldwide studies, 29% participants with screening-detected diabetes had elevated FPG levels, 37% had isolated elevated HbA_1c_ levels, and 31% had an elevation in both parameters.^29^ However, 55% of those with screening-detected diabetes in central and eastern Europe and 45% in high-income Western regions had isolated abnormal FPG levels. Both abnormal FPG and HbA_1c_ levels are associated with increased risk of macrovascular and microvascular disease,^5,30,31^ and there are effective approaches for delaying type 2 diabetes across both categories, with more intensive approaches advised for those at highest risk.^3^ Based on this evidence, by relying on serial FPG measurements, we will have missed some individuals with diabetes in our cohort and possibly overestimated diabetes among individuals who erroneously did not fast for the test. However, our approach was in keeping with prior studies^7,17,18^ and congruent with our study population demographics and the overall practice across REP clinical sites throughout the study period.

### Strengths and Limitations

Our study has some strengths; a major strength is the large cohort size with adequate numbers of events and less than 1% of missing data. Our limited number of variables avoids overfitting our model and ensures sufficient power to test for the associations of, and interactions among, these variables and diabetes risk. To rule out the associations of acute illness with FPG, we excluded blood samples taken during hospitalization. Although several additional variables are associated with diabetes development,^3^ we limited our analyses to sex, BMI, and age, as they are easily measured and consistently recorded in routine clinical encounters, and thus could be easily used to further stratify risk among our study population. There are several assessment tools available for identifying those at risk of developing type 2 diabetes, including the American Diabetes Association risk test, which identifies those who would benefit from testing and does not use any biochemical measures.^3^ This current study assesses diabetes risk among a population of individuals who have already had a baseline FPG test. Biochemical markers, especially FPG level, have been generally shown to improve risk prediction models, but it is always advisable to validate risk scores within the population in which they are intended to be used.^32^

Our study also has several limitations. First, due to the observational design, unmeasured confounding may have affected our risk estimates. With respect to the generalizability of our findings, the sex and racial and ethnic characteristics of Olmsted County residents are similar to those of individuals from the state of Minnesota and the Upper Midwest. Age- and sex-specific mortality rates are similar to those of the entire US.^33^ However, Olmsted County is less racially and ethnically diverse (90.3% vs 75.1% White individuals) and more highly educated (91.1% vs 80.4% high school graduates) than the overall US population.^33^ In this context, we did not include race or ethnicity as variables in our assessment, and future work should include assessing the robustness of our system for diabetes risk stratification in more diverse populations. Within any community-based population, a significant number of people with prediabetes and diabetes are not tested and remain undiagnosed. Such individuals were not captured in our cohort; therefore, it will be biased toward individuals who presented for medical evaluation and had glucose level tested in an outpatient setting, arguably making the findings more relevant to clinical practice. Due to the retrospective study design, to determine progression, individuals were guaranteed at least 1 follow-up FPG test, and survival is assumed in our modeling. We did examine a subset of 6793 individuals who were excluded from our study due to having a single FPG test completed with no follow-up test (eTable 3 in Supplement 1). Compared with our study cohort, those excluded had a lower mean age, BMI, and FPG level, which resulted in a lower overall estimated risk. Finally, as our study focused on identifying those who crossed the FPG threshold to diabetes, we did not assess specific patterns of changes in glucose levels in individuals over time, and we do not have information on those who experienced diabetes remission. There are data to suggest that, among those who develop diabetes, the preceding glucose values are not linear over time^18^; this requires further study. Clinical practice guidelines recommend a second confirmatory test for diabetes for those who are asymptomatic.^5^ Our study did not have this requirement to avoid missing a diabetes diagnosis among individuals who were treated and experienced a lowering of FPG level after the initial elevation was detected. This element of the study design may have resulted in an overestimation of diabetes.

## Conclusions

Overall, this cohort study demonstrates that even within normal ranges, a higher baseline FPG level continues to be a robust marker of future diabetes development. However, an individual’s risk is substantially altered depending on sex, age, and BMI. These variables facilitate an individualized risk-to-benefit assessment when discussing strategies to lower progression to diabetes and associated comorbidities.

## Notes

Supplement 1. **eAppendix.** Age Effect Sensitivity Analyses **eTable 1.** 10-Year Risk Tables for Preferred Additive Model **eTable 2.** Hosmer Lemeshow Comparison of Predicted 10-Year Risk With the Risk Obtained From the Kaplan-Meier Method Within Deciles of Risk **eTable 3.** Baseline Characteristics of Subjects With a Single FPG and No Follow Up Test Who Were Excluded From the Cohort **eFigure 1.** Flow Chart of Participants Included in the Study **eFigure 2.** 10-Year Risk Across Baseline Fasting Plasma Glucose Concentrations, by Body Mass Index Groups for Females (F) (panels A-B) and Males (M) (panels C-D), According to Age Category **eFigure 3.** 10-Year Risk Across Body Mass Index Groups, by Baseline Fasting Plasma Glucose Concentrations for Females (F) (panels A-D) and Males (M) (panels E-H), According to Age Category **eFigure 4.** Hosmer Lemeshow Comparison of Predicted 10-Year Risk With the Risk Obtained From the Kaplan-Meier Method Within Deciles of Risk **eFigure 5.** A Nomogram Assigning a Score to Each Variable Which Can Then be Used to Categorize 10-Year Risk **eFigure 6.** Kaplan Meier Curves for Nomogram Risk Categories

Supplement 2.
Data Sharing Statement
