# Supplementary material for: Baseline Fasting Glucose Level, Age, Sex, and Body Mass Index and the Development of Diabetes in US Adults
Source: JAMA Netw Open. 2025 Jan 23;8(1):e2456067. doi: 10.1001/jamanetworkopen.2024.56067 (PMC11758592; doi:10.1001/jamanetworkopen.2024.56067)
Supplement: Supplement 1. — eAppendix. Age Effect Sensitivity Analyses eTable 1. 10-Year Risk Tables for Preferred Additive Model eTable 2. Hosmer Lemeshow Comparison of Predicted 10-Year Risk With the Risk Obtained From the Kaplan-Meier Method Within Deciles of Risk eTable 3. Baseline Characteristics of Subjects With a Single FPG and No Follow Up Test Who Were Excluded From the Cohort eFigure 1. Flow Chart of Participants Included in the Study eFigure 2. 10-Year Risk Across Baseline Fasting Plasma Glucose Concentrations, by Body Mass Index Groups for Females (F) (panels A-B) and Males (M) (panels C-D), According to Age Category eFigure 3. 10-Year Risk Across Body Mass Index Groups, by Baseline Fasting Plasma Glucose Concentrations for Females (F) (panels A-D) and Males (M) (panels E-H), According to Age Category eFigure 4. Hosmer Lemeshow Comparison of Predicted 10-Year Risk With the Risk Obtained From the Kaplan-Meier Method Within Deciles of Risk eFigure 5. A Nomogram Assigning a Score to Each Variable Which Can Then be Used to Categorize 10-Year Risk eFigure 6. Kaplan Meier Curves for Nomogram Risk Categories [file jamanetwopen-e2456067-s001.pdf]

Supplementary Online Content

Egan AM, Wood-Wentz CM, Mohan S, Bailey KR, Vella A. Baseline fasting glucose level, age, sex, and body mass index and the development of diabetes in US adults. *JAMA Netw Open.* 2025;8(1):e2456067. doi:10.1001/jamanetworkopen.2024.56067

eAppendix. Age Effect Sensitivity Analyses

eTable 1. 10-Year Risk Tables for Preferred Additive Model

eTable 2. Hosmer Lemeshow Comparison of Predicted 10-Year Risk With the Risk Obtained From the Kaplan-Meier Method Within Deciles of Risk

eTable 3. Baseline Characteristics of Subjects With a Single FPG and No Follow Up Test Who Were Excluded From the Cohort

eFigure 1. Flow Chart of Participants Included in the Study

eFigure 2. 10-Year Risk Across Baseline Fasting Plasma Glucose Concentrations, by Body Mass Index Groups for Females (F) (panels A-B) and Males (M) (panels C-D), According to Age Category

eFigure 3. 10-Year Risk Across Body Mass Index Groups, by Baseline Fasting Plasma Glucose Concentrations for Females (F) (panels A-D) and Males (M) (panels E-H), According to Age Category

eFigure 4. Hosmer Lemeshow Comparison of Predicted 10-Year Risk With the Risk Obtained From the Kaplan-Meier Method Within Deciles of Risk

eFigure 5. A Nomogram Assigning a Score to Each Variable Which Can Then be Used to Categorize 10-Year Risk

eFigure 6. Kaplan Meier Curves for Nomogram Risk Categories

This supplementary material has been provided by the authors to give readers additional information about their work.

© 2025 Egan AM et al. *JAMA Network Open.*

eAppendix. Age Effect Sensitivity Analyses

Four models were assessed as follows:

1) A model without an age effect
2) A model with a 4 category age effect (presented in the main manuscript)
3) A model with age and age^2
4) A model with 4 category age effect + age and age^2

The model chi-squares for these 4 models were:

1) No age effect	14 d.f.	3230.048
2) 4-category age effect	17 d.f.	3369.906
3) Age + age^ 2	16 d.f.	3349.163
4) 4-category age + age + age^2	19 d.f.	3377.056

The partial age chisquare using 4-category age effect is 139.859 (3369.906 – 3230.048) and using age + age**2 is 119.116 (3349.163 - 3230.048). The partial chisquare for adding age+age^2 to the categorical age model is 7.150 (3377.056-3369.906) on 2 df, while the partial chisquare for adding categorical age to the age+age^2 model is 27.893 (3377.056-3349.163) on 3 df. This suggests that there is greater improvement of fit, as measured by chi-square, from adding the categorical model to the age+age^2 model than vice versa.

Then, if 10 year risk estimates are compared between the categorical age and continuous age models, the Pearson correlation is 0.99. The two models are virtually interchangeable from a risk prediction point of view. We also compared the individual level effects of age as estimated from the two models (risk ratio of model 2 vs model 1, and risk ratio of model 3 vs model 1), which yielded a Pearson correlation coefficient of 0.87.

Therefore the categorical model, besides being more compatible with the goals of a point score and nomogram, is actually a better fit.

© 2025 Egan AM et al. *JAMA Network Open.*

eTable 1. 10-Year Risk Tables for Preferred Additive Model

Sex	Age Category	Body Mass Index Category	Fasting plasma glucose category	10 year risk (%)
Female	1 <30	1 <18.5	1 <70	22
Female	1 <30	1 <18.5	2 70-79	11
Female	1 <30	1 <18.5	3 80-94	7
Female	1 <30	1 <18.5	4 95-99	9
Female	1 <30	1 <18.5	5 100-104	13
Female	1 <30	1 <18.5	6 105-109	19
Female	1 <30	1 <18.5	7 110-114	27
Female	1 <30	1 <18.5	8 115-119	43
Female	1 <30	1 <18.5	9 120-125	58
Female	1 <30	2 18.5-24	1 <70	10
Female	1 <30	2 18.5-24	2 70-79	4
Female	1 <30	2 18.5-24	3 80-94	3
Female	1 <30	2 18.5-24	4 95-99	4
Female	1 <30	2 18.5-24	5 100-104	6
Female	1 <30	2 18.5-24	6 105-109	8
Female	1 <30	2 18.5-24	7 110-114	12
Female	1 <30	2 18.5-24	8 115-119	21
Female	1 <30	2 18.5-24	9 120-125	30
Female	1 <30	3 25-29	1 <70	13
Female	1 <30	3 25-29	2 70-79	6
Female	1 <30	3 25-29	3 80-94	4
Female	1 <30	3 25-29	4 95-99	5
Female	1 <30	3 25-29	5 100-104	8
Female	1 <30	3 25-29	6 105-109	11
Female	1 <30	3 25-29	7 110-114	16
Female	1 <30	3 25-29	8 115-119	27
Female	1 <30	3 25-29	9 120-125	39
Female	1 <30	4 30-34	1 <70	19
Female	1 <30	4 30-34	2 70-79	9
Female	1 <30	4 30-34	3 80-94	6
Female	1 <30	4 30-34	4 95-99	8
Female	1 <30	4 30-34	5 100-104	12
Female	1 <30	4 30-34	6 105-109	17
Female	1 <30	4 30-34	7 110-114	24
Female	1 <30	4 30-34	8 115-119	39
Female	1 <30	4 30-34	9 120-125	54
Female	1 <30	5 35-39	1 <70	28
Female	1 <30	5 35-39	2 70-79	14
Female	1 <30	5 35-39	3 80-94	9
Female	1 <30	5 35-39	4 95-99	11

© 2025 Egan AM et al. *JAMA Network Open.*

Female	1 <30	5 35-39	5 100-104	17
Female	1 <30	5 35-39	6 105-109	24
Female	1 <30	5 35-39	7 110-114	35
Female	1 <30	5 35-39	8 115-119	53
Female	1 <30	5 35-39	9 120-125	69
Female	1 <30	6 >=40	1 <70	34
Female	1 <30	6 >=40	2 70-79	17
Female	1 <30	6 >=40	3 80-94	11
Female	1 <30	6 >=40	4 95-99	14
Female	1 <30	6 >=40	5 100-104	21
Female	1 <30	6 >=40	6 105-109	29
Female	1 <30	6 >=40	7 110-114	41
Female	1 <30	6 >=40	8 115-119	61
Female	1 <30	6 >=40	9 120-125	77
Female	2 30-54	1 <18.5	1 <70	28
Female	2 30-54	1 <18.5	2 70-79	14
Female	2 30-54	1 <18.5	3 80-94	9
Female	2 30-54	1 <18.5	4 95-99	12
Female	2 30-54	1 <18.5	5 100-104	17
Female	2 30-54	1 <18.5	6 105-109	24
Female	2 30-54	1 <18.5	7 110-114	35
Female	2 30-54	1 <18.5	8 115-119	54
Female	2 30-54	1 <18.5	9 120-125	69
Female	2 30-54	2 18.5-24	1 <70	13
Female	2 30-54	2 18.5-24	2 70-79	6
Female	2 30-54	2 18.5-24	3 80-94	4
Female	2 30-54	2 18.5-24	4 95-99	5
Female	2 30-54	2 18.5-24	5 100-104	8
Female	2 30-54	2 18.5-24	6 105-109	11
Female	2 30-54	2 18.5-24	7 110-114	16
Female	2 30-54	2 18.5-24	8 115-119	27
Female	2 30-54	2 18.5-24	9 120-125	39
Female	2 30-54	3 25-29	1 <70	17
Female	2 30-54	3 25-29	2 70-79	8
Female	2 30-54	3 25-29	3 80-94	5
Female	2 30-54	3 25-29	4 95-99	7
Female	2 30-54	3 25-29	5 100-104	10
Female	2 30-54	3 25-29	6 105-109	15
Female	2 30-54	3 25-29	7 110-114	21
Female	2 30-54	3 25-29	8 115-119	35
Female	2 30-54	3 25-29	9 120-125	49
Female	2 30-54	4 30-34	1 <70	25
Female	2 30-54	4 30-34	2 70-79	12
Female	2 30-54	4 30-34	3 80-94	8

© 2025 Egan AM et al. *JAMA Network Open.*

Female	2 30-54	4 30-34	4 95-99	10
Female	2 30-54	4 30-34	5 100-104	15
Female	2 30-54	4 30-34	6 105-109	22
Female	2 30-54	4 30-34	7 110-114	31
Female	2 30-54	4 30-34	8 115-119	49
Female	2 30-54	4 30-34	9 120-125	64
Female	2 30-54	5 35-39	1 <70	36
Female	2 30-54	5 35-39	2 70-79	18
Female	2 30-54	5 35-39	3 80-94	12
Female	2 30-54	5 35-39	4 95-99	15
Female	2 30-54	5 35-39	5 100-104	22
Female	2 30-54	5 35-39	6 105-109	31
Female	2 30-54	5 35-39	7 110-114	44
Female	2 30-54	5 35-39	8 115-119	64
Female	2 30-54	5 35-39	9 120-125	79
Female	2 30-54	6 >=40	1 <70	43
Female	2 30-54	6 >=40	2 70-79	22
Female	2 30-54	6 >=40	3 80-94	15
Female	2 30-54	6 >=40	4 95-99	18
Female	2 30-54	6 >=40	5 100-104	27
Female	2 30-54	6 >=40	6 105-109	37
Female	2 30-54	6 >=40	7 110-114	51
Female	2 30-54	6 >=40	8 115-119	72
Female	2 30-54	6 >=40	9 120-125	86
Female	3 55-59	1 <18.5	1 <70	36
Female	3 55-59	1 <18.5	2 70-79	18
Female	3 55-59	1 <18.5	3 80-94	12
Female	3 55-59	1 <18.5	4 95-99	15
Female	3 55-59	1 <18.5	5 100-104	22
Female	3 55-59	1 <18.5	6 105-109	31
Female	3 55-59	1 <18.5	7 110-114	44
Female	3 55-59	1 <18.5	8 115-119	64
Female	3 55-59	1 <18.5	9 120-125	79
Female	3 55-59	2 18.5-24	1 <70	17
Female	3 55-59	2 18.5-24	2 70-79	8
Female	3 55-59	2 18.5-24	3 80-94	5
Female	3 55-59	2 18.5-24	4 95-99	7
Female	3 55-59	2 18.5-24	5 100-104	10
Female	3 55-59	2 18.5-24	6 105-109	14
Female	3 55-59	2 18.5-24	7 110-114	21
Female	3 55-59	2 18.5-24	8 115-119	35
Female	3 55-59	2 18.5-24	9 120-125	48
Female	3 55-59	3 25-29	1 <70	22
Female	3 55-59	3 25-29	2 70-79	11

© 2025 Egan AM et al. *JAMA Network Open.*

Female	3 55-59	3 25-29	3 80-94	7
Female	3 55-59	3 25-29	4 95-99	9
Female	3 55-59	3 25-29	5 100-104	13
Female	3 55-59	3 25-29	6 105-109	19
Female	3 55-59	3 25-29	7 110-114	28
Female	3 55-59	3 25-29	8 115-119	44
Female	3 55-59	3 25-29	9 120-125	59
Female	3 55-59	4 30-34	1 <70	32
Female	3 55-59	4 30-34	2 70-79	16
Female	3 55-59	4 30-34	3 80-94	10
Female	3 55-59	4 30-34	4 95-99	13
Female	3 55-59	4 30-34	5 100-104	20
Female	3 55-59	4 30-34	6 105-109	28
Female	3 55-59	4 30-34	7 110-114	39
Female	3 55-59	4 30-34	8 115-119	59
Female	3 55-59	4 30-34	9 120-125	75
Female	3 55-59	5 35-39	1 <70	45
Female	3 55-59	5 35-39	2 70-79	23
Female	3 55-59	5 35-39	3 80-94	16
Female	3 55-59	5 35-39	4 95-99	20
Female	3 55-59	5 35-39	5 100-104	29
Female	3 55-59	5 35-39	6 105-109	39
Female	3 55-59	5 35-39	7 110-114	53
Female	3 55-59	5 35-39	8 115-119	74
Female	3 55-59	5 35-39	9 120-125	88
Female	3 55-59	6 >=40	1 <70	52
Female	3 55-59	6 >=40	2 70-79	28
Female	3 55-59	6 >=40	3 80-94	19
Female	3 55-59	6 >=40	4 95-99	24
Female	3 55-59	6 >=40	5 100-104	35
Female	3 55-59	6 >=40	6 105-109	46
Female	3 55-59	6 >=40	7 110-114	62
Female	3 55-59	6 >=40	8 115-119	82
Female	3 55-59	6 >=40	9 120-125	93
Female	4 >=60	1 <18.5	1 <70	48
Female	4 >=60	1 <18.5	2 70-79	26
Female	4 >=60	1 <18.5	3 80-94	17
Female	4 >=60	1 <18.5	4 95-99	21
Female	4 >=60	1 <18.5	5 100-104	31
Female	4 >=60	1 <18.5	6 105-109	42
Female	4 >=60	1 <18.5	7 110-114	57
Female	4 >=60	1 <18.5	8 115-119	78
Female	4 >=60	1 <18.5	9 120-125	90
Female	4 >=60	2 18.5-24	1 <70	24

© 2025 Egan AM et al. *JAMA Network Open.*

Female	4 >=60	2 18.5-24	2 70-79	12
Female	4 >=60	2 18.5-24	3 80-94	7
Female	4 >=60	2 18.5-24	4 95-99	10
Female	4 >=60	2 18.5-24	5 100-104	14
Female	4 >=60	2 18.5-24	6 105-109	20
Female	4 >=60	2 18.5-24	7 110-114	30
Female	4 >=60	2 18.5-24	8 115-119	47
Female	4 >=60	2 18.5-24	9 120-125	62
Female	4 >=60	3 25-29	1 <70	31
Female	4 >=60	3 25-29	2 70-79	15
Female	4 >=60	3 25-29	3 80-94	10
Female	4 >=60	3 25-29	4 95-99	13
Female	4 >=60	3 25-29	5 100-104	19
Female	4 >=60	3 25-29	6 105-109	27
Female	4 >=60	3 25-29	7 110-114	38
Female	4 >=60	3 25-29	8 115-119	57
Female	4 >=60	3 25-29	9 120-125	73
Female	4 >=60	4 30-34	1 <70	44
Female	4 >=60	4 30-34	2 70-79	23
Female	4 >=60	4 30-34	3 80-94	15
Female	4 >=60	4 30-34	4 95-99	19
Female	4 >=60	4 30-34	5 100-104	28
Female	4 >=60	4 30-34	6 105-109	38
Female	4 >=60	4 30-34	7 110-114	52
Female	4 >=60	4 30-34	8 115-119	73
Female	4 >=60	4 30-34	9 120-125	87
Female	4 >=60	5 35-39	1 <70	58
Female	4 >=60	5 35-39	2 70-79	33
Female	4 >=60	5 35-39	3 80-94	22
Female	4 >=60	5 35-39	4 95-99	28
Female	4 >=60	5 35-39	5 100-104	39
Female	4 >=60	5 35-39	6 105-109	52
Female	4 >=60	5 35-39	7 110-114	68
Female	4 >=60	5 35-39	8 115-119	87
Female	4 >=60	5 35-39	9 120-125	96
Female	4 >=60	6 >=40	1 <70	66
Female	4 >=60	6 >=40	2 70-79	39
Female	4 >=60	6 >=40	3 80-94	27
Female	4 >=60	6 >=40	4 95-99	33
Female	4 >=60	6 >=40	5 100-104	47
Female	4 >=60	6 >=40	6 105-109	60
Female	4 >=60	6 >=40	7 110-114	76
Female	4 >=60	6 >=40	8 115-119	92
Female	4 >=60	6 >=40	9 120-125	98

© 2025 Egan AM et al. *JAMA Network Open.*

Male	1 <30	1 <18.5	1 <70	28
Male	1 <30	1 <18.5	2 70-79	14
Male	1 <30	1 <18.5	3 80-94	9
Male	1 <30	1 <18.5	4 95-99	11
Male	1 <30	1 <18.5	5 100-104	17
Male	1 <30	1 <18.5	6 105-109	24
Male	1 <30	1 <18.5	7 110-114	34
Male	1 <30	1 <18.5	8 115-119	52
Male	1 <30	1 <18.5	9 120-125	68
Male	1 <30	2 18.5-24	1 <70	12
Male	1 <30	2 18.5-24	2 70-79	6
Male	1 <30	2 18.5-24	3 80-94	4
Male	1 <30	2 18.5-24	4 95-99	5
Male	1 <30	2 18.5-24	5 100-104	7
Male	1 <30	2 18.5-24	6 105-109	11
Male	1 <30	2 18.5-24	7 110-114	16
Male	1 <30	2 18.5-24	8 115-119	26
Male	1 <30	2 18.5-24	9 120-125	38
Male	1 <30	3 25-29	1 <70	17
Male	1 <30	3 25-29	2 70-79	8
Male	1 <30	3 25-29	3 80-94	5
Male	1 <30	3 25-29	4 95-99	6
Male	1 <30	3 25-29	5 100-104	10
Male	1 <30	3 25-29	6 105-109	14
Male	1 <30	3 25-29	7 110-114	21
Male	1 <30	3 25-29	8 115-119	34
Male	1 <30	3 25-29	9 120-125	48
Male	1 <30	4 30-34	1 <70	24
Male	1 <30	4 30-34	2 70-79	12
Male	1 <30	4 30-34	3 80-94	8
Male	1 <30	4 30-34	4 95-99	10
Male	1 <30	4 30-34	5 100-104	15
Male	1 <30	4 30-34	6 105-109	21
Male	1 <30	4 30-34	7 110-114	30
Male	1 <30	4 30-34	8 115-119	48
Male	1 <30	4 30-34	9 120-125	63
Male	1 <30	5 35-39	1 <70	35
Male	1 <30	5 35-39	2 70-79	18
Male	1 <30	5 35-39	3 80-94	12
Male	1 <30	5 35-39	4 95-99	15
Male	1 <30	5 35-39	5 100-104	22
Male	1 <30	5 35-39	6 105-109	30
Male	1 <30	5 35-39	7 110-114	43
Male	1 <30	5 35-39	8 115-119	63

© 2025 Egan AM et al. *JAMA Network Open.*

Male	1 <30	5 35-39	9 120-125	78
Male	1 <30	6 >=40	1 <70	41
Male	1 <30	6 >=40	2 70-79	22
Male	1 <30	6 >=40	3 80-94	14
Male	1 <30	6 >=40	4 95-99	18
Male	1 <30	6 >=40	5 100-104	26
Male	1 <30	6 >=40	6 105-109	36
Male	1 <30	6 >=40	7 110-114	50
Male	1 <30	6 >=40	8 115-119	71
Male	1 <30	6 >=40	9 120-125	85
Male	2 30-54	1 <18.5	1 <70	35
Male	2 30-54	1 <18.5	2 70-79	18
Male	2 30-54	1 <18.5	3 80-94	12
Male	2 30-54	1 <18.5	4 95-99	15
Male	2 30-54	1 <18.5	5 100-104	22
Male	2 30-54	1 <18.5	6 105-109	31
Male	2 30-54	1 <18.5	7 110-114	43
Male	2 30-54	1 <18.5	8 115-119	63
Male	2 30-54	1 <18.5	9 120-125	79
Male	2 30-54	2 18.5-24	1 <70	16
Male	2 30-54	2 18.5-24	2 70-79	8
Male	2 30-54	2 18.5-24	3 80-94	5
Male	2 30-54	2 18.5-24	4 95-99	6
Male	2 30-54	2 18.5-24	5 100-104	10
Male	2 30-54	2 18.5-24	6 105-109	14
Male	2 30-54	2 18.5-24	7 110-114	21
Male	2 30-54	2 18.5-24	8 115-119	34
Male	2 30-54	2 18.5-24	9 120-125	47
Male	2 30-54	3 25-29	1 <70	22
Male	2 30-54	3 25-29	2 70-79	10
Male	2 30-54	3 25-29	3 80-94	7
Male	2 30-54	3 25-29	4 95-99	9
Male	2 30-54	3 25-29	5 100-104	13
Male	2 30-54	3 25-29	6 105-109	19
Male	2 30-54	3 25-29	7 110-114	27
Male	2 30-54	3 25-29	8 115-119	43
Male	2 30-54	3 25-29	9 120-125	58
Male	2 30-54	4 30-34	1 <70	32
Male	2 30-54	4 30-34	2 70-79	16
Male	2 30-54	4 30-34	3 80-94	10
Male	2 30-54	4 30-34	4 95-99	13
Male	2 30-54	4 30-34	5 100-104	19
Male	2 30-54	4 30-34	6 105-109	27
Male	2 30-54	4 30-34	7 110-114	39

© 2025 Egan AM et al. *JAMA Network Open.*

Male	2 30-54	4 30-34	8 115-119	58
Male	2 30-54	4 30-34	9 120-125	74
Male	2 30-54	5 35-39	1 <70	44
Male	2 30-54	5 35-39	2 70-79	23
Male	2 30-54	5 35-39	3 80-94	15
Male	2 30-54	5 35-39	4 95-99	19
Male	2 30-54	5 35-39	5 100-104	28
Male	2 30-54	5 35-39	6 105-109	39
Male	2 30-54	5 35-39	7 110-114	53
Male	2 30-54	5 35-39	8 115-119	74
Male	2 30-54	5 35-39	9 120-125	87
Male	2 30-54	6 >=40	1 <70	52
Male	2 30-54	6 >=40	2 70-79	28
Male	2 30-54	6 >=40	3 80-94	19
Male	2 30-54	6 >=40	4 95-99	23
Male	2 30-54	6 >=40	5 100-104	34
Male	2 30-54	6 >=40	6 105-109	46
Male	2 30-54	6 >=40	7 110-114	61
Male	2 30-54	6 >=40	8 115-119	81
Male	2 30-54	6 >=40	9 120-125	92
Male	3 55-59	1 <18.5	1 <70	44
Male	3 55-59	1 <18.5	2 70-79	23
Male	3 55-59	1 <18.5	3 80-94	15
Male	3 55-59	1 <18.5	4 95-99	19
Male	3 55-59	1 <18.5	5 100-104	28
Male	3 55-59	1 <18.5	6 105-109	39
Male	3 55-59	1 <18.5	7 110-114	53
Male	3 55-59	1 <18.5	8 115-119	74
Male	3 55-59	1 <18.5	9 120-125	87
Male	3 55-59	2 18.5-24	1 <70	21
Male	3 55-59	2 18.5-24	2 70-79	10
Male	3 55-59	2 18.5-24	3 80-94	7
Male	3 55-59	2 18.5-24	4 95-99	8
Male	3 55-59	2 18.5-24	5 100-104	13
Male	3 55-59	2 18.5-24	6 105-109	18
Male	3 55-59	2 18.5-24	7 110-114	27
Male	3 55-59	2 18.5-24	8 115-119	42
Male	3 55-59	2 18.5-24	9 120-125	58
Male	3 55-59	3 25-29	1 <70	28
Male	3 55-59	3 25-29	2 70-79	14
Male	3 55-59	3 25-29	3 80-94	9
Male	3 55-59	3 25-29	4 95-99	11
Male	3 55-59	3 25-29	5 100-104	17
Male	3 55-59	3 25-29	6 105-109	24

© 2025 Egan AM et al. *JAMA Network Open.*

Male	3 55-59	3 25-29	7 110-114	34
Male	3 55-59	3 25-29	8 115-119	53
Male	3 55-59	3 25-29	9 120-125	69
Male	3 55-59	4 30-34	1 <70	40
Male	3 55-59	4 30-34	2 70-79	20
Male	3 55-59	4 30-34	3 80-94	13
Male	3 55-59	4 30-34	4 95-99	17
Male	3 55-59	4 30-34	5 100-104	25
Male	3 55-59	4 30-34	6 105-109	35
Male	3 55-59	4 30-34	7 110-114	48
Male	3 55-59	4 30-34	8 115-119	69
Male	3 55-59	4 30-34	9 120-125	84
Male	3 55-59	5 35-39	1 <70	54
Male	3 55-59	5 35-39	2 70-79	29
Male	3 55-59	5 35-39	3 80-94	20
Male	3 55-59	5 35-39	4 95-99	25
Male	3 55-59	5 35-39	5 100-104	36
Male	3 55-59	5 35-39	6 105-109	48
Male	3 55-59	5 35-39	7 110-114	63
Male	3 55-59	5 35-39	8 115-119	83
Male	3 55-59	5 35-39	9 120-125	94
Male	3 55-59	6 >=40	1 <70	62
Male	3 55-59	6 >=40	2 70-79	35
Male	3 55-59	6 >=40	3 80-94	24
Male	3 55-59	6 >=40	4 95-99	30
Male	3 55-59	6 >=40	5 100-104	42
Male	3 55-59	6 >=40	6 105-109	56
Male	3 55-59	6 >=40	7 110-114	71
Male	3 55-59	6 >=40	8 115-119	89
Male	3 55-59	6 >=40	9 120-125	97
Male	4 >=60	1 <18.5	1 <70	58
Male	4 >=60	1 <18.5	2 70-79	32
Male	4 >=60	1 <18.5	3 80-94	22
Male	4 >=60	1 <18.5	4 95-99	27
Male	4 >=60	1 <18.5	5 100-104	39
Male	4 >=60	1 <18.5	6 105-109	51
Male	4 >=60	1 <18.5	7 110-114	67
Male	4 >=60	1 <18.5	8 115-119	86
Male	4 >=60	1 <18.5	9 120-125	95
Male	4 >=60	2 18.5-24	1 <70	30
Male	4 >=60	2 18.5-24	2 70-79	15
Male	4 >=60	2 18.5-24	3 80-94	10
Male	4 >=60	2 18.5-24	4 95-99	12
Male	4 >=60	2 18.5-24	5 100-104	18

© 2025 Egan AM et al. *JAMA Network Open.*

Male	4 >=60	2 18.5-24	6 105-109	26
Male	4 >=60	2 18.5-24	7 110-114	37
Male	4 >=60	2 18.5-24	8 115-119	56
Male	4 >=60	2 18.5-24	9 120-125	72
Male	4 >=60	3 25-29	1 <70	38
Male	4 >=60	3 25-29	2 70-79	20
Male	4 >=60	3 25-29	3 80-94	13
Male	4 >=60	3 25-29	4 95-99	16
Male	4 >=60	3 25-29	5 100-104	24
Male	4 >=60	3 25-29	6 105-109	33
Male	4 >=60	3 25-29	7 110-114	46
Male	4 >=60	3 25-29	8 115-119	67
Male	4 >=60	3 25-29	9 120-125	82
Male	4 >=60	4 30-34	1 <70	53
Male	4 >=60	4 30-34	2 70-79	29
Male	4 >=60	4 30-34	3 80-94	19
Male	4 >=60	4 30-34	4 95-99	24
Male	4 >=60	4 30-34	5 100-104	35
Male	4 >=60	4 30-34	6 105-109	47
Male	4 >=60	4 30-34	7 110-114	62
Male	4 >=60	4 30-34	8 115-119	82
Male	4 >=60	4 30-34	9 120-125	93
Male	4 >=60	5 35-39	1 <70	68
Male	4 >=60	5 35-39	2 70-79	40
Male	4 >=60	5 35-39	3 80-94	28
Male	4 >=60	5 35-39	4 95-99	34
Male	4 >=60	5 35-39	5 100-104	48
Male	4 >=60	5 35-39	6 105-109	62
Male	4 >=60	5 35-39	7 110-114	77
Male	4 >=60	5 35-39	8 115-119	93
Male	4 >=60	5 35-39	9 120-125	98
Male	4 >=60	6 >=40	1 <70	76
Male	4 >=60	6 >=40	2 70-79	48
Male	4 >=60	6 >=40	3 80-94	34
Male	4 >=60	6 >=40	4 95-99	41
Male	4 >=60	6 >=40	5 100-104	56
Male	4 >=60	6 >=40	6 105-109	70
Male	4 >=60	6 >=40	7 110-114	84
Male	4 >=60	6 >=40	8 115-119	96
Male	4 >=60	6 >=40	9 120-125	99

© 2025 Egan AM et al. *JAMA Network Open.*

eTable 2. Hosmer Lemeshow Comparison of Predicted 10-Year Risk With the Risk Obtained From the Kaplan-Meier Method Within Deciles of Risk

Decile of risk	Predicted 10-year risk (%)	Observed 10-year risk (%)	Chi-square
1	45.4	44.6	0.55
2	21.9	22.8	0.90
3	15.5	16.6	1.84
4	12.2	11.1	2.59
5	9.8	9.8	0.01
6	8.0	7.8	0.16
7	6.7	7.0	0.38
8	5.3	5.4	0.02
9	4.5	4.3	0.21
10	3.5	2.6	7.92

© 2025 Egan AM et al. *JAMA Network Open.*

eTable 3. Baseline Characteristics of Subjects With a Single FPG and No Follow Up Test Who Were Excluded From the Cohort

**N**		**6793**
**Sex**	**Female**	4083 (60.1)
	**Male**	2710 (39.9)
**Age (years)**		36.4 (12.6)
**Age Category (years)**	**<30**	2600 (38.3)
	**30-54.9**	3439 (50.6)
	**55 – 59.9**	350 (5.2)
	**≥ 60**	404 (5.9)
**BMI (kg/m^2)**		27.8 (6.7)
**BMI Category (kg/m^2)**	**<18.5**	159 (2.3)
	**18.5 – 24.9**	2407 (35.4)
	**25.0 – 29.9**	2193 (32.3)
	**30.0 – 34.9**	1140 (16.8)
	**35 – 39.9**	516 (7.6)
	**≥ 40**	378 (5.6
**FPG (mg/dL)**		92.4 (9.5)
**FPG Category (mg/dL)***	**<80**	480 (7.1)
	**80-99**	4986 (73.4)
	**100-109**	969 (14.3)
	**110-125**	358 (5.3)

Data are n (%), mean (SD)
BMI: body mass index; FPG: fasting plasma glucose
**To convert to mmol/L, multiply by 0.0555*

© 2025 Egan AM et al. *JAMA Network Open.*

eFigure 1. Flow Chart of Participants Included in the Study

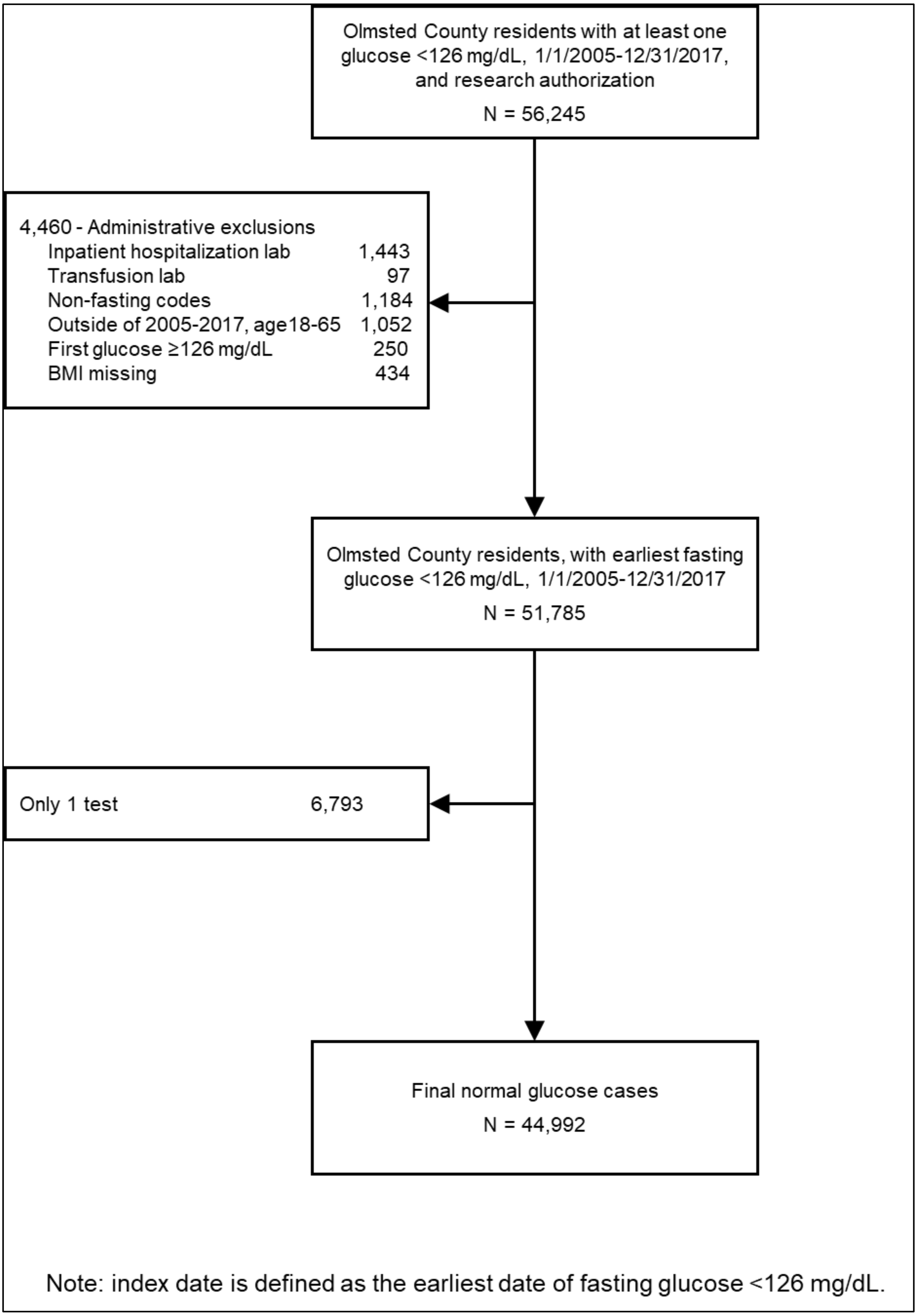

© 2025 Egan AM et al. *JAMA Network Open.*

eFigure 2. 10-Year Risk Across Baseline Fasting Plasma Glucose Concentrations, by Body Mass Index Groups for Females (F) (panels A-B) and Males (M) (panels C-D), According to Age Category

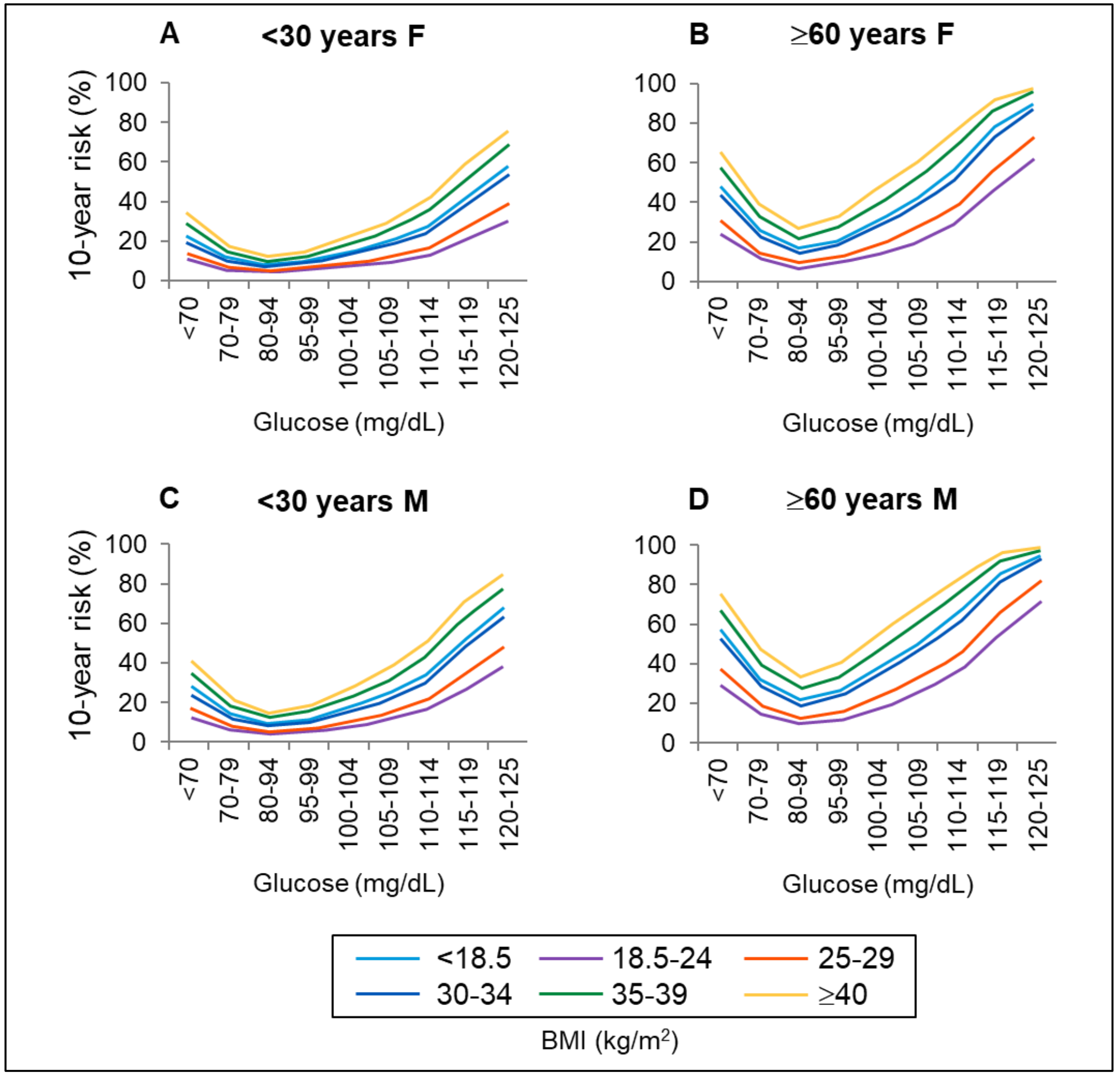

© 2025 Egan AM et al. *JAMA Network Open.*

eFigure 3. 10-Year Risk Across Body Mass Index Groups, by Baseline Fasting Plasma Glucose Concentrations for Females (F) (panels A-D) and Males (M) (panels E-H), According to Age Category

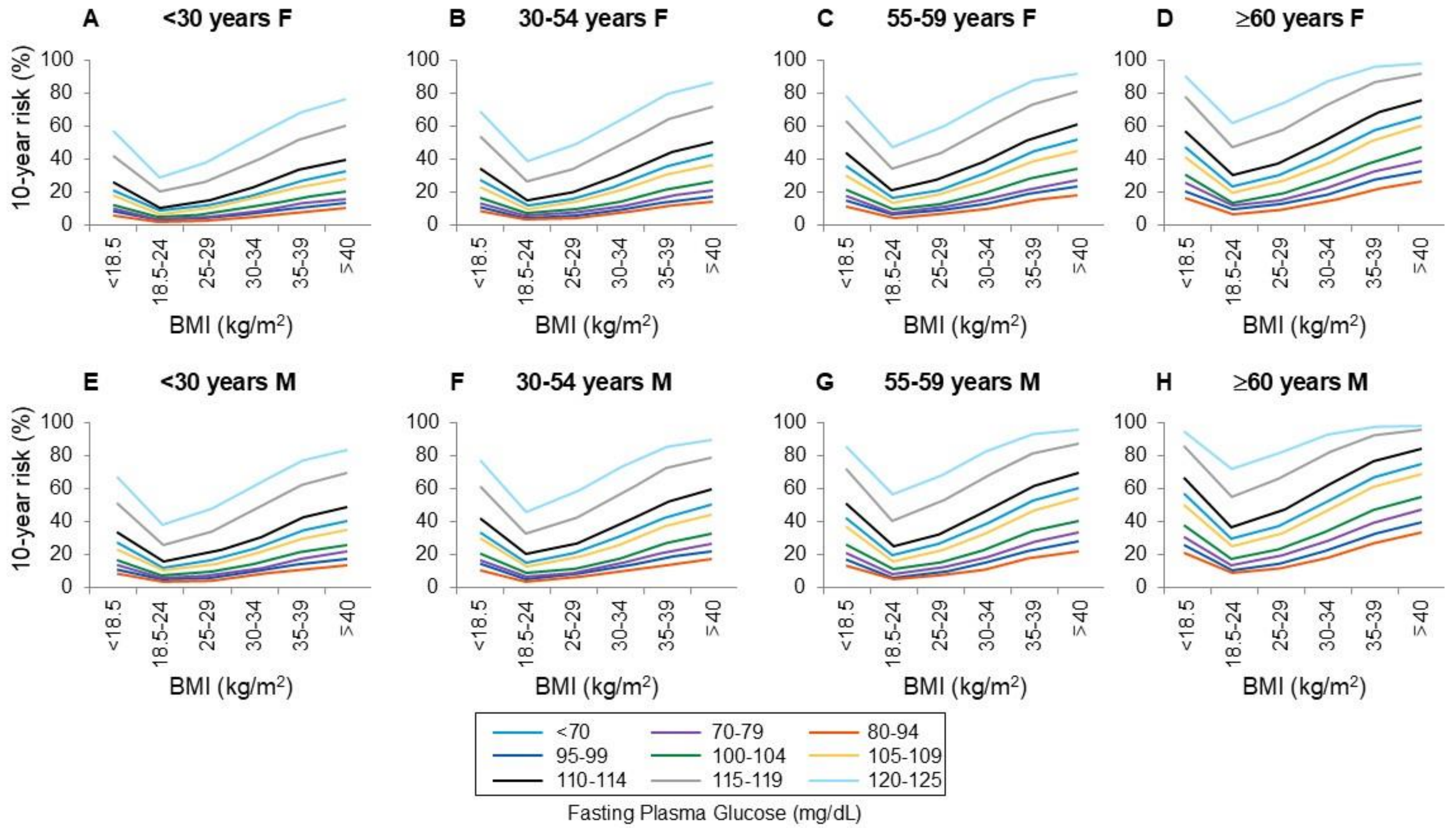

© 2025 Egan AM et al. *JAMA Network Open.*

eFigure 4. Hosmer Lemeshow Comparison of Predicted 10-Year Risk With the Risk Obtained From the Kaplan-Meier Method Within Deciles of Risk

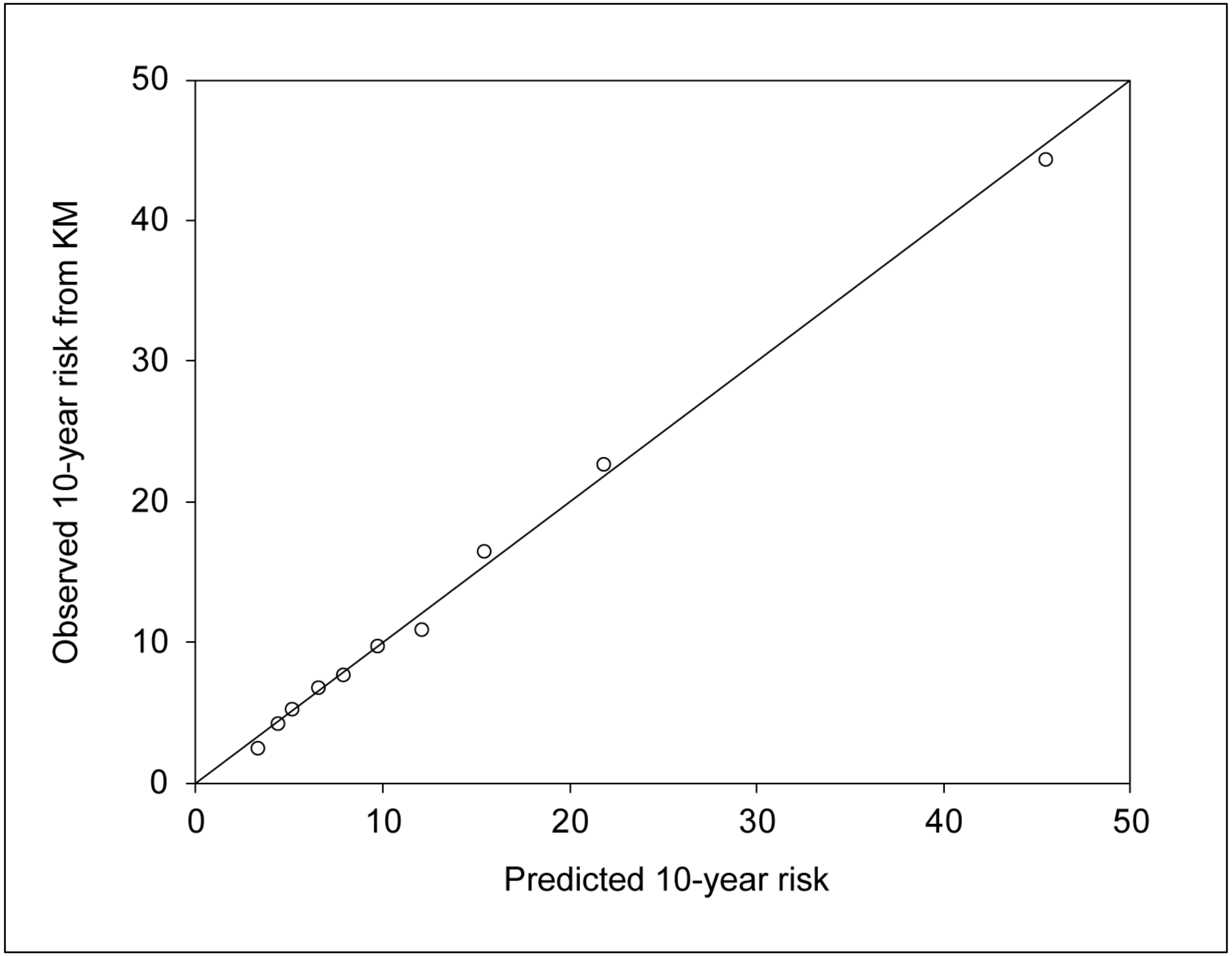

© 2025 Egan AM et al. *JAMA Network Open.*

eFigure 5. A Nomogram Assigning a Score to Each Variable Which Can Then be Used to Categorize 10-Year Risk

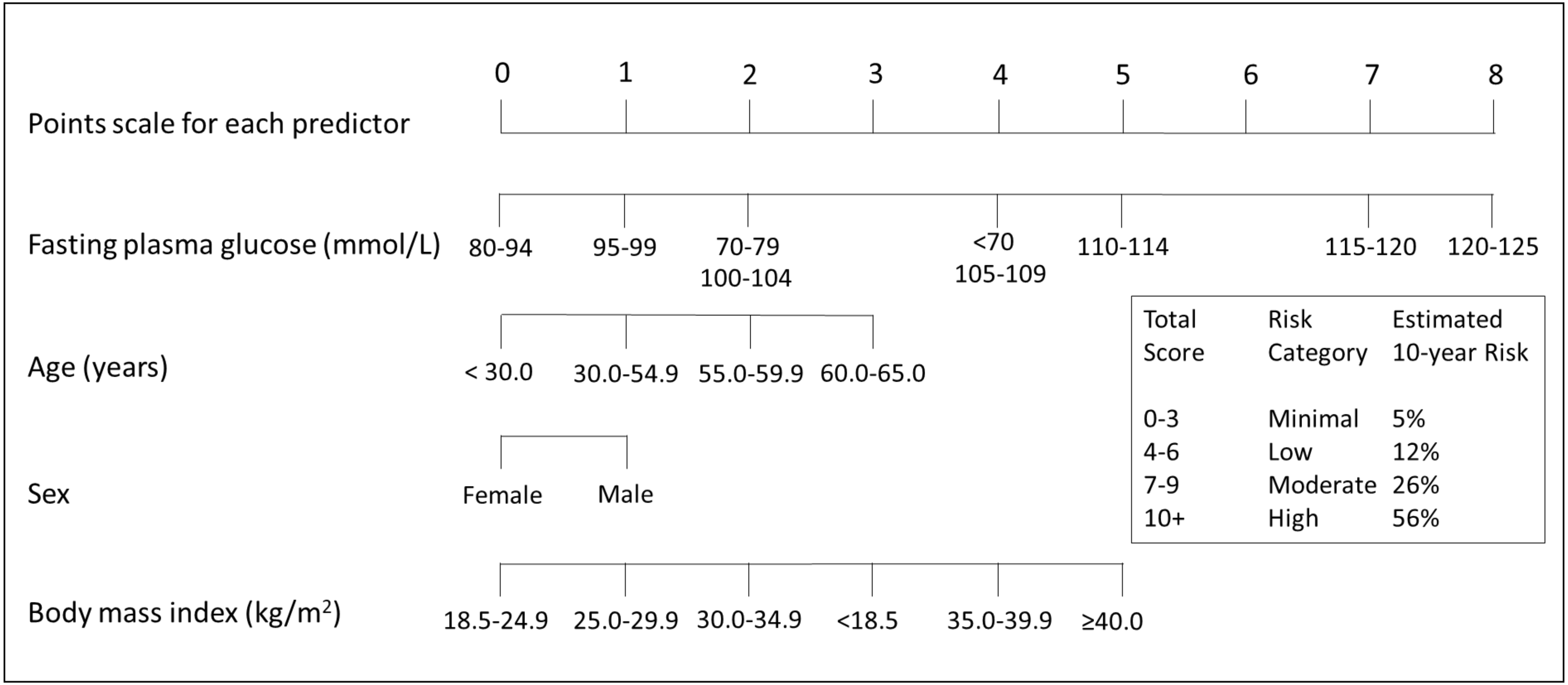

© 2025 Egan AM et al. *JAMA Network Open.*

eFigure 6. Kaplan Meier Curves for Nomogram Risk Categories

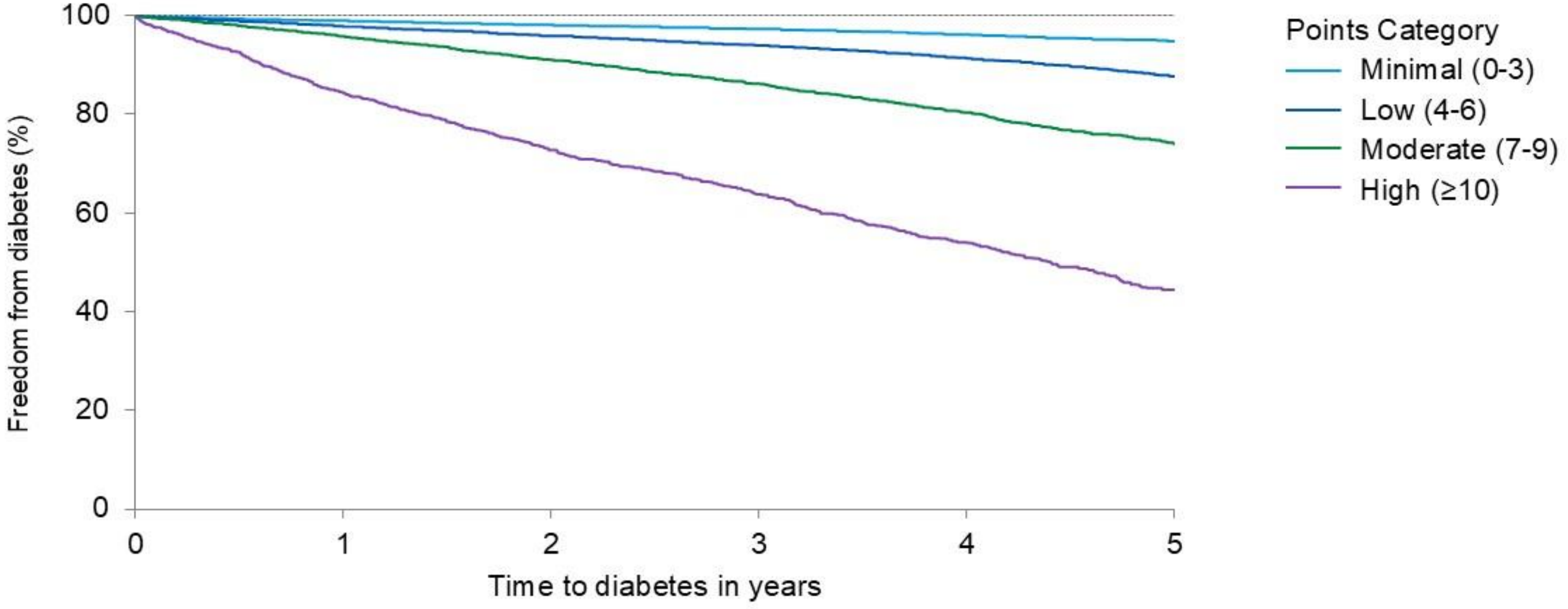

© 2025 Egan AM et al. *JAMA Network Open.*
