# Supplementary material for: Baseline Fasting Glucose Level, Age, Sex, and Body Mass Index and the Development of Diabetes in US Adults
Source: JAMA Netw Open. 2025 Jan 23;8(1):e2456067. doi: 10.1001/jamanetworkopen.2024.56067 (PMC11758592; doi:10.1001/jamanetworkopen.2024.56067)
Supplement: Supplement 2. — Data Sharing Statement [file jamanetwopen-e2456067-s002.pdf]

Data Sharing Statement

Egan. Baseline Fasting Glucose Level, Age, Sex, and Body Mass Index and the Development of Diabetes in US Adults. *JAMA Netw Open*. Published January 23, 2025. doi:10.1001/jamanetworkopen.2024.56067

Data
Data available: Yes
Data types: Deidentified participant data
How to access data: egan.aoife@mayo.edu
When available: With publication

Supporting Documents
Document types: None

Additional Information
Who can access the data: researchers whose proposed use of the data has been approved
Types of analyses: For any purpose
Mechanisms of data availability: With investigator support, after approval of a proposal, and with a signed data access agreement
